# The PL6-Family Plasmids of *Haloquadratum* Are Virus-Related

**DOI:** 10.3389/fmicb.2018.01070

**Published:** 2018-05-23

**Authors:** Mike Dyall-Smith, Friedhelm Pfeiffer

**Affiliations:** ^1^Computational Biology Group, Max Planck Institute of Biochemistry, Martinsried, Germany; ^2^Department of Veterinary Biosciences, Faculty of Veterinary and Agricultural Sciences, University of Melbourne, Parkville, VIC, Australia

**Keywords:** archaea, haloarchaea, halobacteria, *Hqr. walsbyi*, plasmid, PL6, virus

## Abstract

Plasmids PL6A and PL6B are both carried by the C23^T^ strain of the square archaeon *Haloquadratum walsbyi*, and are closely related (76% nucleotide identity), circular, about 6 kb in size, and display the same gene synteny. They are unrelated to other known plasmids and all of the predicted proteins are cryptic in function. Here we describe two additional PL6-related plasmids, pBAJ9-6 and pLT53-7, each carried by distinct isolates of *Haloquadratum walsbyi* that were recovered from hypersaline waters in Australia. A third PL6-like plasmid, pLTMV-6, was assembled from metavirome data from Lake Tyrell, a salt-lake in Victoria, Australia. Comparison of all five plasmids revealed a distinct plasmid family with strong conservation of gene content and synteny, an average size of 6.2 kb (range 5.8–7.0 kb) and pairwise similarities between 61–79%. One protein (F3) was closely similar to a protein carried by betapleolipoviruses while another (R6) was similar to a predicted AAA-ATPase of His 1 halovirus (His1V_gp16). Plasmid pLT53-7 carried a gene for a FkbM family methyltransferase that was not present in any of the other plasmids. Comparative analysis of all PL6-like plasmids provided better resolution of conserved sequences and coding regions, confirmed the strong link to haloviruses, and showed that their sequences are highly conserved among examples from *Haloquadratum* isolates and metagenomic data that collectively cover geographically distant locations, indicating that these genetic elements are widespread.

## Introduction

*Haloquadratum walsbyi* is a thin, square-shaped haloarchaeon belonging to the family *Haloferacaceae* (Burns et al., 2007; Gupta et al., 2015). It is extremely halophilic and commonly inhabits salt-saturated environments where it is often the dominant prokaryote (Tully et al., 2015) but is notoriously difficult to isolate in the laboratory. Even though first described in 1980 (Walsby, 1980), it was not until 2004 that cultivation success was reported (Bolhuis et al., 2004; Burns et al., 2004). In 2011, the genome sequence of the type strain, *Hqr. walsbyi* C23^T^, revealed the presence of two, small (6 kb), closely related plasmids, PL6A and PL6B (Burns et al., 2007; Dyall-Smith et al., 2011). These showed little similarity to other known sequences except for one pair of predicted proteins (Hqrw_6002 and its ortholog Hqrw_7002), which closely resembled proteins encoded by a wide variety of haloarchaea and that were located nearby halovirus-related gene clusters. Later, it was found that genes similar to Hqrw_6002 were always carried by members of the betapleolipovirus group of haloviruses, such as HRPV-3 (Liu et al., 2015), but the function of this protein remains unknown. A second pair of plasmid proteins (Hqrw_6005 and its ortholog Hqrw_7005) was weakly related to a different halovirus protein, HisV_gp16 (ORF 16) from the lemon-shaped (or spindle-shaped) halovirus His1 (*Salterprovirus* genus). Although the function of HisV_gp16 remains unknown it is not present among the structural proteins of the virus (Pietila et al., 2013; Hong et al., 2015). Both PL6 proteins Hqrw_6002 and Hqrw_6005 are predicted to contain winged-helix DNA binding domains, and Hqrw_6005 is also predicted to contain an AAA-ATPase domain (Dyall-Smith et al., 2011).

Since 2010 (Oh et al., 2010), no new isolates of *Haloquadratum* have been described, probably because of the perceived difficulty involved in cultivation, and while metagenomic studies have allowed partial genomes of this genus to be assembled without the need for cultivation (Tully et al., 2015) there have been no additional examples of *Haloquadratum*-derived, completely sequenced PL6-like plasmids available in the sequence databases (GenBank, accessed February 2018). Their significance is however, becoming more evident, as a recent metagenomic study suggested that PL6-like plasmids may be present in upward of 32–40% of the *Haloquadratum* population of Lake Tyrrell (LT; Tully et al., 2015).

Although not formally reported in publications, additional strains of *Hqr. walsbyi* have been isolated and deposited in culture collections or sent to other laboratories. Examples include strains Bajool9 (also called BJCX4_extB9.1) and Cry7_14, that were isolated as part of the study described in (Oh et al., 2010) and deposited with the Japan Collection of Microbes under the accessions JCM 15065 and JCM 15557, respectively. Another three strains, including the LT53-19 strain described in the current study, were isolated from Lake Tyrell water in 2009 (MDS, unpublished) and sent to the laboratory of Eric Allen (University of California, San Diego, United States). Strain Bajool9 was mentioned in our 2011 study as carrying a PL6-like plasmid of about 6 kb in size (Dyall-Smith et al., 2011), but sequence data were not reported. Further sequence data from PL6-like plasmids would allow better resolution of the likely encoded genes, provide insight into their function and diversity, and help to establish the true nature of these replicons, in particular their relationship to known haloviruses and their proviruses.

In this study, we present and compare the sequences of PL6-related plasmids from *Haloquadratum* strains Bajool9 and LT53-19, as well as a plasmid assembled from LT metavirome data collected by Emerson et al. (Emerson et al., 2012, 2013). These new plasmids were closely related to PL6A/B, shared similar genes and gene synteny, and displayed specific relationships to known haloviruses.

## Materials and Methods

### Isolates and Sources

Strains of *Haloquadratum* were isolated from salt water samples using the extinction dilution method described in (Burns et al., 2004; Dyall-Smith, 2009). *Hqr. walsbyi* strain LT53-19 was isolated from a LT brine sample (taken on January 4, 2009) provided to MDS by Dr. Karla Heidelberg (University of Southern California, United States). Strain LT53-19 was sent to, and is available from, Dr. Eric Allen (University of California, San Diego, United States). The Bajool9 strain of *Haloquadratum* was isolated in the MDS laboratory in 2007 as part of the study reported in (Oh et al., 2010), and was mentioned later in (Dyall-Smith et al., 2011) and deposited in the Japan Collection of Microorganisms (accession JCM 15065). It was recovered from a brine sample taken from a crystallizer pond of the Bajool saltern in Queensland, Australia. The coordinates of LT and the Bajool saltern are given in **Table 1**.

**Table 1:** Details of plasmids described in this study.

Plasmid	Length (nt)	GC%	Accession	Publication	Host strain/source
PL6A	6129	51	FR746101	(Dyall-Smith et al., 2011)	*Hqr. walsbyi* C23^T^ DSM 16854 Isolated from Cheetham saltern, Victoria Origin 38° 9′45.45″S 144°25′25.51″E
PL6B	6056	52	FR746102	(Dyall-Smith et al., 2011)	*Hqr. walsbyi* C23^T^ DSM 16854 Isolated from Cheetham saltern, Victoria Origin 38° 9′45.45″S 144°25′25.51″E
pBAJ9-6	6213	53	LT984491	This study	*Hqr. walsbyi* Bajool9 JCM 15065 Isolated from Bajool saltern, Queensland (Oh et al., 2010) Origin 23°35′10.11″S 150°49′42.08″E
pLT53-7	7045	51	LT984489	This study	*Hqr. walsbyi* LT53-19. Isolated from Lake Tyrrell, 2009. Origin 35°19′10.02″S 142°47′1.61″E
pLTMV-6	5884	53	LT991975	This study	Assembled from Lake Tyrrell metavirome^a^


*^*a*^Bioproject PRJNA81851, Lake Tyrrell 2007-2010, viral concentrate samples. Data (SRR402039, SRR402041-47) published in (Emerson et al., 2013).*

### Plasmid Sequencing

The sequences of plasmids PL6A and PL6B (accessions FR746101 and FR746102) were used to design consensus PCR primers for detecting and amplifying related plasmids in other strains of *Haloquadratum*. Amplified products were sequenced [ABI PRISM BigDye terminator method, performed at the Core Facility of the Max-Planck-Institute of Biochemistry (Dyall-Smith et al., 2011)] and the resulting sequences used to design primers for further rounds of PCR/sequencing or primer-walking on purified plasmid, until plasmid closure. The sequences have been deposited under accessions LT984489 and LT984491.

### pLTMV-6 Plasmid Sequence Assembled From Metavirome Data

The LT metavirome data (accessions SRR402039 and SRR402041-47) were downloaded from the GenBank sequence read archive (SRA), imported into Geneious (v10.2.3) and the reads mapped to the pLT53-7 plasmid sequence (Geneious mapper). Reads mapping to that plasmid were then de novo reassembled (Geneious assembler) and the longest contig (which also had the greatest read coverage) was used for further rounds of mapping against the metavirome reads in order to extend the contig ends. The process was repeated for several rounds until closure. The final sequence was checked manually at every base against the mapped reads, in order to identify and correct errors. Annotation was performed in the Geneious (v10.2.3) environment using plasmids PL6A, PL6B and pLT53-7 for reference. The sequence has been deposited under accession LT991975.

### Search for CRISPR Spacers Matching Plasmids

Spacer matches to PL6-related plasmids were sought from multiple sources; (1) the CRISPR finder database of spacers^1^; (2) the prokaryotic spacers collection produced as part of the study by (Shmakov et al., 2017); (3) spacers extracted from metagenomic data available at the SRA^2^ using the crass v0.3.12 software (Skennerton et al., 2013), i.e., metagenomes were searched for using keywords (hypersaline, saltern, LT, crystallizer, and salt lake) and the appropriate data downloaded and processed for spacers on a laptop computer. The extracted spacers were then transformed into a BLAST database (in Geneious) and used to search for matches to PL6-family plasmid sequences. The crass software also outputs the direct repeats (DR) associated with the discovered spacers.

### Bioinformatics Analyses

#### Phylogenetic Tree Reconstructions

Plasmid nucleotide sequences were aligned in the Geneious aligner and phylogenetic tree reconstructions performed using the MrBayes algorithm within Geneious v10.2.3. Protein sequences matching those of PL6-family plasmids were identified by BLASTP searches at NCBI, downloaded into Geneious, aligned using the Geneious aligner, edited manually to remove duplicates and incomplete sequences, and trees inferred using neighbor-joining (PAUP^∗^) with 100 bootstrap repetitions.

#### RNA and Protein Structure Predictions

RNA and protein structure predictions used the RNAfold^3^, RNAstructure^4^, and I-Tasser^5^ webservers.

## Results

### Haloquadratum Isolates and Plasmid Sequences

Two novel Australian isolates of *Haloquadratum* (**Table 1**) were each found to carry a PL6-like plasmid, designated pBAJ9-6 and pLT53-7. One isolate (LT53-19) was recovered in 2009 from LT (Victoria) and the other (Bajool9) in 2007 from a saltern crystallizer pond near Bajool (Queensland). An agarose gel profile of the Bajool9 plasmid has been previously reported [see Figure 2A of (Dyall-Smith et al., 2011)]. The sequences of both plasmids were determined and compared to the PL6 plasmids (PL6A, PL6B) of *Hqr. walsbyi* C23^T^ (Dyall-Smith et al., 2011), an isolate recovered in 2004 from the Cheetham saltern in Geelong, Victoria (Burns et al., 2004; Burns et al., 2007). The sites of isolation of all these strains are geographically well separated, e.g., Geelong, on the south coast of Victoria, is 348 km from LT, and 1727 km from Bajool in Queensland. The coordinates of all sites are given in **Table 1**.

**FIGURE 1:**
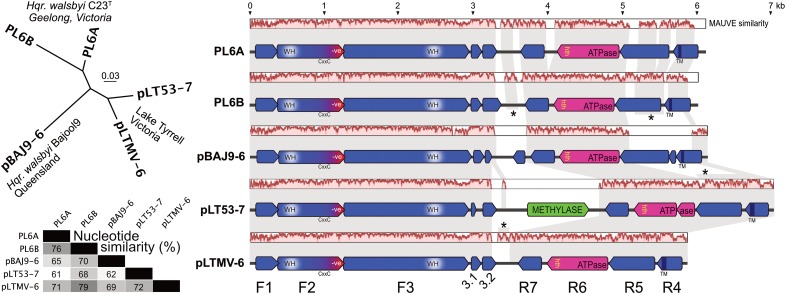
Comparison of PL6-family plasmids. **(A)** phylogenetic tree reconstruction (MrBayes) of the five plasmids. Isolate and location details are shown near the corresponding plasmids. Scale bar represents 0.03 estimated changes per site. **(B)** percentage nucleotide similarity of the aligned plasmids. **(C)** gene arrangements and similarity of PL6-family plasmids. Annotated coding sequences are shown as horizontal arrows, above which are nucleotide similarity plots based on a MAUVE alignment, with background gray shading used to connect regions of similar sequence. Asterisks denote vertical connections (of sequence similarity) between different plasmid levels where one or two of the intervening plasmids show very low sequence similarity to the plasmids above and below, as indicated by the MAUVE similarity plots (e.g., between the R5 regions of PL6B and pLT53-7, where the intervening pBAJ9-6 plasmid shows little similarity to either). The major, common protein coding sequences are labeled F1–F3 and R4–R7 at bottom. Scale shown at top, in kb.

The general properties of pBAJ9-6 and pLT53-7 are given in **Table 1**, along with the previously published details of plasmids PL6A and PL6B. Also shown in **Table 1** is a PL6-like plasmid, pLTMV-6, that was reconstructed from LT metavirome data (see section “Materials and Methods” and below). The five plasmids were of comparable size (5.8 – 7.0 kb) and their GC contents spanned a narrow range (51–53%) but were significantly above that of the chromosome of *Hqr. walsbyi* (47.8% GC, (Dyall-Smith et al., 2011)). Multiple alignment using the MAUVE algorithm (Darling et al., 2004) (**Figure 1**) gave pairwise similarities of 61–79% (average, 69%), with the region from nt 1 to about 3.2 kb showing a higher average sequence similarity (84%; as indicated by the MAUVE plots), compared to the sequences from 3.2 kb to the right end (54%). This characteristic was noted previously for PL6A and PL6B (Dyall-Smith et al., 2011). The split into two subregions is also obvious from the coding potential. Two sets of protein-coding genes are transcribed in opposite orientation from an intergenic region which traverses the point where the plasmid sequences have been linearized. There are three strongly conserved and closely spaced protein coding genes encoded on the forward strand (F1–F3) in the first half of these plasmids. All plasmids have four protein coding genes encoded on the reverse strand (R4–R7), covering the second half of the plasmid.

Phylogenetic tree reconstructions (MrBayes) based on nucleotide alignments produced clades that corresponded to the host strain and/or geographical origin (**Figure 1**, upper left panel), i.e., plasmids PL6A/PL6B of *Hqr. walsbyi* C23^T^ formed one clade, and the LT plasmids formed another clade, while the plasmid from the Bajool saltern in Queensland (Oh et al., 2010) branched as a distinct lineage.

While the average GC% for all plasmids was 52%, plots of GC% (Supplementary Figure S1, blue lines) revealed a consistently higher than average value (55%) across the first ∼3.2 kb, a region encompassing genes F1–F3. Immediately following this is a distinctly AT-rich central region from ∼3.2 kb to the end of R7 (41–43% GC), and from R7 to the start of R4 the values fluctuated but overall were around the average for the entire plasmid (51%). Of the various nucleotide skew plots, GC profile plots (Gao and Zhang, 2006) gave good discrimination between different plasmid regions (Supplementary Figure S1), with minima near or within the F1 gene rising steadily to maxima at about 3.1 kb followed by a steep drop in the AT-rich central, intergenic region between the C-termini of F3 and R7. After this, in most cases, there is a gentler drop to the end. The annotated gene diagrams below the x-axes show how these plots correspond to the major gene blocks. pLT53-7 differed slightly from the others in having a very regular rise and fall around the maximum followed by a gentle rise (rather than a drop) to the end. The altered pattern is at least in part due to an extended AT-rich central region (GC% = 37.8) that corresponds with a methylase gene found between F1 and R7 only in this plasmid, and transcribed in the same direction as the F1–F3 protein encoding genes.

### Sequence Matches to Hypersaline Metagenomes and Metaviromes

Publicly available metagenomic sequences from hypersaline lakes were searched to detect sequences matching those of the PL6-family plasmids and were readily detected in the large datasets available for LT (Australia), Santa Pola (Spain) and Lake Meyghan (Iran) (**Table 2**). The highest counts were found in the LT data with LT metavirome sequences giving higher counts compared to LT metagenome data. However, it should be noted that this increase (2–3 fold) is paralleled by an even higher increase in total read number (∼14-fold). The plasmid pLT53-7 matched the most metavirome reads (>14,000), and this plasmid originated from a *Haloquadratum* strain isolated from the same lake. The abundance of metavirome reads allowed the assembly of a 5.88 kb, circular, PL6-family plasmid from this data (see section Materials and methods), that was designated pLTMV-6 and included in the following analyses.

**Table 2:** Hypersaline environment metagenomic reads matching PL6-family plasmids.

Origin	Sequence reads matching plasmids:^a^					
				
	PL6A	PL6B	pBAJ9-6	pLT53-7	Accession^b^	Total reads	Reference
Lake Tyrrell (Australia)	4412	4195	4011	4958	SRX2875668-9	2.3 × 10^6^	(Podell et al., 2014)
Santa Pola (Spain)	547	571	545	591	SRR979792, SRR944625	2.5 × 10^6^	(Santos et al., 2010)
Lake Meyghan (Iran)	11	61	58	30	ERR1739732-3, ERR1742999-3002	72.2 × 10^6^	(Naghoni et al., 2017)
**Metavirome**			
Lake Tyrrell (Australia)	9996	12964	10706	14085	SRX117679-86	32.6 × 10^6^	(Emerson et al., 2013)


*^*a*^BLASTN searches using a threshold *E* ≤ 10^-*10*^. ^*b*^sequence read archive files at www.ncbi.nlm.nih.gov/sra, or www.ebi.ac.uk/ena.*

### Conserved and Novel Coding Sequences

To simplify description, the major protein coding genes shown in **Figure 1** have been numbered F1–F3 for genes encoded on the forward strand from left to right, and R4–R7 for genes encoded on the reverse strand from right to left (**Figure 1**, bottom level). The characteristics of the predicted proteins are summarized in **Table 3** and protein alignments are given in Supplementary Figures S2, S3.

**Table 3:** Comparison of predicted major, conserved proteins of PL6-family plasmids.

CDS	Length^a^ (average)	% Identity (average)	pI (average)	GenBank matches^b^	Conserved Domains^c^	Comments
F1	97–100 (99)	38–71 (54)	9.4–10.3 (9.7)	0	0	basic protein (while F2–R7 are acidic)
F2	296–298 (297)	77–91 (85)	4.47–4.71 (4.5)	0	Winged helix (WH) (∼aa 1–77)	CxxC motif, and strongly negative (acidic) c-terminus
F3	567–581 (570)	80–96 (88)	4.79–4.91 (4.9)	>100	Winged helix (WH) (near c-terminus)	Similar to betapleolipovirus protein, related proviruses, and an uncultured halovirus contig (GU735118); possible replicase
R7	108–112 (109)	71–88 (80)	4.09–4.25 (4.2)	0	0	
R6	275–281 (278)	71–92 (84)	4.83–4.98 (4.9)	5	AAA-ATPase (∼aa 35–150) Winged helix (∼aa 244–265)	Similar to some Halobacteria, two metavirome contigs (GU735304, GU735174), and halovirus His1V_gp16. Putative packaging ATPase.
R5	208–223 (214)	21–89 (47)	4.41–5.01 (4.7)	0	0	c-terminal sequence (7 aa) identical in all R5 proteins
R4	109–120 (115)	17–84 (30)	4.30–5.25 (4.7)	0	transmembrane (TM) domain (∼aa 99–120)	


*^*a*^Length in amino acids. ^*b*^BLASTN algorithm against non-redundant nucleotide collection at blast.ncbi.nlm.nih.gov. ^*c*^Conserved domains found by CD-Search (NCBI) or by InterProScan.*

#### Proteins F1 to F3

The genes for proteins F1–F3 are oriented in the same direction, are present in the same order in all plasmids and have overlapping stop/start codons, suggesting they are transcribed together as an operon.

Protein F1 was not annotated previously for PL6A and PL6B (Dyall-Smith et al., 2011) but is now included as the ortholog pair Hqrw_6000 and Hqrw_7000. The F1 coding sequences were annotated in all five plasmids because of their consistent presence, and that the inferred proteins were of similar size and sequence. Their average isoelectric pH is unusually high (9.7) for haloarchaeal proteins, which are generally acidic (Dyall-Smith et al., 2011). The pI values of the other plasmid proteins were 4 – 5 (**Table 3**), a figure close to the average pI of *Hqr. waslbyi* proteins, which is 5.1 (Dyall-Smith et al., 2011). No significant matches could be found in GenBank (BLASTP/TBLASTN, accessed Feb. 2018), and no conserved protein domains (NCBI, CD-search) were detected. The predicted protein structures (I-Tasser) did not show any clear resemblance to other proteins (data not shown). All haloarchaeal genomes, including *Haloquadratum*, encode a small proportion of basic proteins (pI values ≥9), and many of these are functionally important, such as ribosomal and membrane-transport proteins (Dyall-Smith et al., 2011). Pleolipovirus genomes also encode a small proportion of highly basic (pI ≥ 11) proteins, including the (alphapleolipovirus) HRPV-3 orf6, HGPV-1 and HRPV-6 orf9 proteins, and the (betapleolipovirus) HHPV-1 and HHPV-2 orf8 proteins (Pietila et al., 2016). None of these have been detected in purified virus particles and their functions remain unknown.

The F2 proteins were well conserved in sequence (77 – 91% aa identity) but showed no significant match to other proteins in GenBank (BLASTP/TBLASTN; accessed February, 2018). All were predicted to possess a winged-helix DNA binding domain near the N-terminus (aa 1-77), and all contained a Cys-x-x-Cys motif near the C-terminus as well as a highly negatively charged carboxy-terminal sequence. The first two features suggest a DNA binding function and the highly acidic c-terminus is reminiscent of SSB proteins such as those of *E. coli* (Inoue et al., 2011) and phage T7 (Marintcheva et al., 2008).

The F3 proteins are relatively long (567–581 aa), closely similar in sequence (80–96% aa identity), contain a predicted winged helix DNA binding domain, and strongly match the sequence of ORF9 protein of the betapleolipovirus HRPV-3 (44–48% aa identity), as well as to the corresponding proteins of related haloviruses such as HGPV-1 and SNJ1 (Liu et al., 2015). BLASTP searches of the GenBank database returned >100 matches from various genera of haloarchaea, consistent with the frequent presence of proviruses or virus remnants of the pleolipovirus family in the genomes of these species (Pietila et al., 2016). For example, a close match to the F3 protein of PL6A occurs in *Natrinema ejinorense* (CP557_12410; 46% aa identity), and inspection of the neighboring genes revealed a 15.7 kb provirus-like element bounded by tRNA-Ala at one end, and a pHK2-like integrase (CP557_12365) with an adjacent 14 bp direct repeat of tRNA-Ala at the other end. In between are genes encoding halovirus related proteins, including a phiH-like repressor (CP557_12370), proteins similar to HGTV-1 ORF128 (CP557_12420), and many pleolipovirus-related proteins, such as His2-like (gammapleolipovirus) major capsid protein (CP557_12380), HRPV-3 (betapleolipovirus) VP1-like protein (CP557_12375) and HHPV-3 (alphapleolipovirus) proteins 5 and 6 (CP557_12390, CP557_12395) and HHPV-4 (betapleolipovirus) ORF16 (CP557_12400). This element appears to be an integrated provirus, and highlights both the strong connection between F3 proteins and haloviruses, and the fluid nature of halovirus gene composition.

A characteristic shared by the all PL6-family F3 genes, HRPV-3 ORF9 and two other virus homologs is that they are all preceded by an upstream protein-coding gene that overlaps at the start codon, and in all but one case the overlapping upstream genes encode proteins contain one or more CxxC motifs, as seen in the PL6-family F2 proteins. This was also true in the *Nnm. ejinorense* provirus-like element described before. Phylogenetic tree reconstructions clustered F3 proteins together as a distinct clade, separate from betapleolipovirus and chromosomally encoded F3-relatives. (Supplementary Figure S4), indicating they represent a distinct lineage. The function of F3 remains to be established but the existing evidence suggests it may be a novel type of replicase (Krupovic et al., 2017).

Two short CDS were detected just downstream of F3, and were designated F3.1 and F3.2 (**Figure 1**) but the evidence supporting them is less persuasive than F1–F3. The inferred F3.1 proteins are short (≤50 aa) and predicted to have high pI values (8–11.4) but are well conserved (72–100%). Previously not annotated for PL6A and PL6B (Dyall-Smith et al., 2011) they have now designated as Hqrw_6002A and Hqrw_7002A, and represent orthologs. The F3.2 proteins vary in length (34–89 aa) but all begin with an identical 19 aa sequence. In strain C23^T^ they are represented by the ortholog pair Hqrw_6003 and Hqrw_7003. No conserved protein domains or significant matches to the sequence databases (NCBI) were detected for either the F3.1 or F3.2 proteins.

#### Proteins R4 to R7

The genes for these proteins are in the opposite orientation to the F1–F3 genes (**Figure 1**). They occur in the same order in all plasmids and are closely spaced (30 nt average intergenic distance) but usually do not overlap (1 case in 15). Except for the R6 protein of pLT53-7, the inferred protein sequences and sizes within each protein group are strongly conserved, and the predicted functional domains of proteins R4 and R6 are retained in all cases. Only the R6 proteins show database matches but the number is low; a few matches occur in haloarchaeal genomes, two in putative halovirus contigs and one in halovirus His1 (Bath and Dyall-Smith, 1998; Bath et al., 2006). The His1 protein (His1V_gp16) has been shown not to be a virus structural protein (Pietila et al., 2013; Hong et al., 2015), and the predicted ATPase and winged-helix DNA binding domains could indicate it is a packaging ATPase for the viral genome. Regarding R4 proteins, all are predicted to have a transmembrane domain near the c-terminus but do not have any detectable signal sequence, indicating they are likely to be membrane anchored. Evidence that the R4 proteins of PL6A/B (Hqrw_6007, Hqrw_7007) are present in cell membrane preparations was reported earlier (Dyall-Smith et al., 2011).

The sequence of the R6 gene of pLT53-7 contained a stop codon at nt 5745-7, within the CDS, and the split protein sequence is indicated in **Figure 1** by two, consecutive, pink-shaded arrows. Close comparison with the other R6 proteins and their genes pinpointed where a single base insertion between nt 5889 – 5890 would recover a complete R6 CDS with a protein sequence very similar to the others (see Supplementary Figure S2). The Sanger sequencing reads across this region were clear in both directions, and examination of the many LT metagenome reads that mapped to this gene showed a large proportion had the same sequence, which would result in a stop codon at the same position found in pLT53-7. It may be a pseudogene but a -1 programmed ribosomal frame-shift near to nt 5889 would also produce a full-length R5 protein, and close inspection of this region detected a sequence from nt 5882–5889 of pLT53-7 that is similar to -1 translational slippage sites (Yu et al., 2011) such as those previously reported in haloviruses, e.g., HCTV-1 (Sencilo et al., 2013). A nearby stem-loop structure is commonly positioned just downstream of ribosomal frameshifting sites (Yu et al., 2011) and the sequence at nt 5847–5876 can form a stem-loop with a binding energy of -19.6 kcal/mol.

#### FkmB Family Methylase Gene

Only pLT53-7 carries this gene, and nucleotide alignments with the other PL6-family plasmids indicated that this coding sequence is part of a longer, unique, AT-rich region (nt 3249–4744, ∼1.5 kb) separating F3 from R7. The inferred protein is predicted to contain a methyltransferase FkbM domain (E-value, 10^-12^) but close homologs were not found in the GenBank database (accessed February, 2018). An FkbM domain methylase found in the *Sinorhizobium* phage PhiLM21 genome was described as a ‘moron’, or extra gene (Dziewit et al., 2014).

Following the method of (Porter et al., 2005), *Hqr. walsbyi* C23^T^ cell supernatants were treated with PEG to concentrate any viruses present, and the resuspended pellet examined by negative-stain EM. No virus-like particles were detected (data not shown).

### Representation of PL6-Family Sequences in CRISPR Spacers of Haloarchaea

The CRISPR spacers of available haloarchaeal genomes and metagenomes were searched (see section Materials and Methods) for matches to PL6-family plasmids. Three spacers were found that showed moderate nucleotide similarity (**Table 4**); one to each of the F2 and F3 genes, and the third overlapped the start of F3.2, a small gene downstream of F3. Two spacers were from metagenomic data of Lake Meyghan, and one was from the genome of *Haloferax alexandrinus* (Khelaifia et al., 2017). The direct repeats (DR) flanking the Lake Meyghan metagenome spacers were most similar to those found in *Halogeometricum* and *Natrinema* (not shown). Predicted translations of the spacers were compared with the predicted protein sequences of the putative targets of the PL6 plasmids, and the alignments (**Table 4**, right column) showed a good correspondence. In case 1, the proposed start and stop codons (shown as their complement) are underlined in the nucleotide alignment, and are identical, i.e., an amber (TAG) stop codon, indicated by •_A_, two nucleotides from a GTG start codon.

**Table 4:** CRISPR spacers of haloarchaea with similarity to PL6-family plasmids.

No.	Spacer versus Plasmid Alignment	Comments/Translation^a^
1	DR: GCTTCAACCCCACAACGGGTTCATCTGGAAC	Spans stop/start of consecutive ORFs
	G14SP2056: TTTTGGTTGTGTGCCCTTAGGCACACCTCTAACGGG (L.Meyghan MG)	R •_A_ MCLRAHNQ – SP2056
	PL6A/B: TTTTGATGTTGTGCCCTTAGGCACACCCCTAACAGC (nt 3158-3123)	C •_A_ MCLRAQHQ – PL6A/B F3.2
	^∗∗∗∗∗^^∗^^∗^^∗^^∗∗^^∗∗∗∗∗^^∗^^∗^^∗∗^^∗^^∗^^∗∗^^∗∗∗∗∗^^∗^	^∗^^∗^^∗∗∗∗^^∗^
2	DR: GCTTCGACCCCACAAGGGTCCGTCTGAAAC	within F3 CDS;
	G21SP4836: CCACCGCCTCCCCGTGGAGTGTAAGCACTACTACGA (L.Meyghan MG)	HRLPVECKHYY – SP4836
	pBAJ9-6: TCATCAACTCCCCGTCGAGTGCAAGCACTACTATGC (nt 2059-2094)	HQLPVECKHYY – protein F3
	^∗^^∗^^∗^^∗^^∗∗∗^^∗^^∗^^∗^^∗^^∗^^∗^^∗^^∗∗^^∗∗∗^^∗^^∗∗^^∗∗∗∗∗^^∗^	^∗^ : ^∗∗^^∗∗∗∗^^∗∗∗^
3	DR: GTTTCAGACGAACCAAGCTGGTGTTGAAGC	within F2 CDS;
	Hfx/SP24: GCCTCGTCCTCGTCCTCGTCGTCGCTCTCGA-CTTCG LK053000.1	EVESDDEDEDE – SP24
	PL6B: TCGTCGTCCTCGTCCTCGTCGTCGCCGTTGTTCTTCT (nt 1245-1209)	KNNGDDEDEDD – protein F2
	^∗^^∗^^∗^^∗∗∗^^∗∗∗^^∗∗∗^^∗∗∗^^∗∗∗^^∗∗∗^^∗∗^^∗^^∗^^∗∗∗^^∗^	: : . ^∗∗^^∗∗^^∗∗^ .


*^*a*^Amber stop, TAG, ⋅_*A*_; symbols under alignment (^∗^:.) indicate identical, similar and weakly similar residues, respectively (based on Gonnet PAM 250 matrix).*

### Regulatory Elements and Conserved Intergenic Regions

Plasmid sequences were examined for ribosome binding site sequences but good matches to the consensus (GGAGGTGA) were not found near annotated start codons except for the F3 gene, where the sequence GGAGGcGA was consistently located 4 nt upstream of the F3 start codon (i.e., within F2). These results are consistent with previous studies showing that SD sequences are rarely used in *Haloferax*, and possibly restricted to internal genes of operons (Kramer et al., 2014). Haloarchaeal promoter motifs were also sought, particularly in the region between genes F1 and R4 where outward transcription of the F1–F3 genes and R4–R7 genes is likely to initiate. The consensus promoter motif SRnnRnnnTTWW (Babski et al., 2016) detected 9–13 potential sites per plasmid but only two were both intergenic and conserved in position across all plasmids. These two promoter motifs were overlapping and oriented in opposite directions, and found embedded in a highly conserved sequence (designated CIS I) within the F1 – R4 intergenic region (Supplementary Figure S5A). Nearby is another highly conserved intergenic sequence (CIS II), which could be involved in gene regulation of the F1–F3 operon.

At the other end of the F1 – R4 intergenic region, the R4 CDS was seen to begin with an ATG start codon in every case, and this was supported by LT metagenomic data that mapped to pLT53-7 or pLTMV-6 (data not shown). Leaderless transcripts are known to be very common in haloarchaea and the great majority (94%) of these use an ATG start codon (Brenneis et al., 2007; Hering et al., 2009; Babski et al., 2016), while GTG codons appear to be reserved for poorly expressed genes (Babski et al., 2016). Inspection of the upstream sequences of the R4 genes for potential promoter motifs centered at -27 to -28 (Babski et al., 2016) relative to the A of the initiator revealed inverted repeats centered around -16 to -25 and containing AT-motifs of 4–5 nt in length that were present in every plasmid (red arrows in Supplementary Figure S5A). The R4 proximal inverted repeats may represent regulatory sequences that encompass promoter motifs that are less closely similar to the *Haloferax* consensus. Additional conserved intergenic sequences (CIS III, IV, and V) were found midway between the F3 and R7 genes and immediately downstream of the R6 coding sequence (Supplementary Figures S5B,C).

## Discussion

The five PL6-family plasmids compared in this study originate from three geographically well separated sites in Australia yet retain a strikingly similar organizational pattern, with two blocks of outwardly directed, tightly-spaced genes that cover most of the circular 6–7 kb of dsDNA. Between the origins of these two gene blocks is a relatively short sequence containing two highly conserved intergenic sequences (CIS) and R4 proximal inverted repeats (PIRs). If CIS I and the R4 PIRs are shown to contain promoters, then they could initiate transcription of F1 and R4, which could progress in both directions until termination beyond F3.2 in one direction, and beyond R7 in the other. This would provide a means of tightly controlling gene expression, and is similar to the transcriptional strategy described recently for pHRDV-1 (Chen et al., 2014), a 13 kb pleolipovirus-like plasmid with a gene organization resembling that of the PL6 plasmids. The lack of consensus ribosome binding sites in the PL6-family plasmids is not surprising, as previous studies have shown that haloarchaea frequently use leaderless mRNAs, and even in those transcripts with 5′UTRs the use of a RBS appears to be unnecessary (Kramer et al., 2014). There is evidence that RBS may be used within multigene transcripts, possibly as ribosome pause sites to enhance translational coupling (Kramer et al., 2014; Babski et al., 2016), which would be consistent with the analysis here, where a consensus RBS motif was found just upstream of the F3 CDS.

The F1–F3 gene block is well conserved between the five plasmids, at both the nucleotide and predicted protein levels, and has a higher than average GC%. The oppositely directed R4–R7 genes are less well conserved but retain a consistent gene order. The region between the ends of these two gene blocks is AT-rich, more variable in sequence and less clearly resolved with regard to its genetic content but presumably includes termination sites for the two major transcripts and is sufficiently flexible that it can incorporate extra genes, as evidenced by the DNA methylase gene found in pLT53-7.

Of the seven proteins (F1–R7) carried by all plasmids only F3 and R6 matched sequences present in the standard databases (GenBank), and both proteins showed strong similarity to haloviruses or halovirus-like genomic loci found in a variety of haloarchaeal species. In particular, the F3 protein is closely related to a putative replicase carried by betapleolipoviruses (Krupovic et al., 2017). The PL6-family plasmids show further resemblances to betapleolipoviruses, as both have circular dsDNA, are of similar size and share a similar pattern of gene organization (Pietila et al., 2016). A strong connection to haloviruses was also seen in the large number of matches to metavirome sequences from LT, enabling the assembly of a complete PL6-like plasmid (pLTMV-6).

The PL6 family plasmids may well be provirus forms of a novel halovirus group, and it is often difficult to distinguish between the two types of genetic elements. For example, plasmid pHH205 was later found to be the proviral form of SNJ1 virus (Zhang et al., 2012), and the temperate virus phiCh1 is very similar in sequence to plasmid pNMAG03 (Siddaramappa et al., 2012). Indeed, phiCh1 was isolated after the spontaneous lysis of *Natrialba magadii* (which carries pNMAG03) (Witte et al., 1997). Other, unresolved cases include plasmids pHRDV-1 (Chen et al., 2014) and pHK2 (Roine et al., 2010) which closely resemble known pleolipoviruses. CRISPR spacers usually target foreign DNA (Shmakov et al., 2017), including viruses and plasmids, so the potential spacer matches found in this study do not help to resolve whether the PL6 plasmids are proviruses.

The mode of replication of these plasmids remains uncertain as no DNA polymerase gene has yet been identified and sequence motifs typical of host chromosome replication were not detected. If the F3 protein is indeed a replicase then a rolling-circle mode of replication (Holmes et al., 1995; Chen et al., 2016) is likely, as has been demonstrated recently for the temperate sphaerolipovirus SNJ1 (Wang et al., 2018). An intriguing feature of PL6A and PL6B is their stable co-occurrence in the same host strain (Dyall-Smith et al., 2011), while pLT53-7 and pBAJ9-6 occur alone. Closely related plasmids are usually incompatible in that they eventually purify out in a population of host cells so that only one type remains. Perhaps the 24% difference in nucleotide sequence between PL6A and PL6B is sufficient for them to be compatible, and so coexist stably in the cell population. Alternatively, if they are incompatible, release of low levels of virus coupled with cell carriage of one or the other provirus as a plasmid could allow co-persistence and continual infection of any plasmid-free cells arising in the population.

## Author Contributions

MD-S and FP both designed and undertook this study, and wrote the manuscript.

## Conflict of Interest Statement

The authors declare that the research was conducted in the absence of any commercial or financial relationships that could be construed as a potential conflict of interest.

## Footnotes

1 crispr.i2bc.paris-saclay.fr/

2 www.ncbi.nlm.nih.gov/sra

3 rna.tbi.univie.ac.at/

4 rna.urmc.rochester.edu/

5 zhanglab.ccmb.med.umich.edu/I-TASSER/
