# Supplementary material for: The PL6-Family Plasmids of Haloquadratum Are Virus-Related
Source: Front Microbiol. 2018 May 23;9:1070. doi: 10.3389/fmicb.2018.01070 (PMC5974055; doi:10.3389/fmicb.2018.01070)
Supplement: Supplementary file 1 [file Data_Sheet_1.DOCX]

**Supplementary Figures and Tables.**

**Figure S1. GC profiles of PL6-family plasmids**


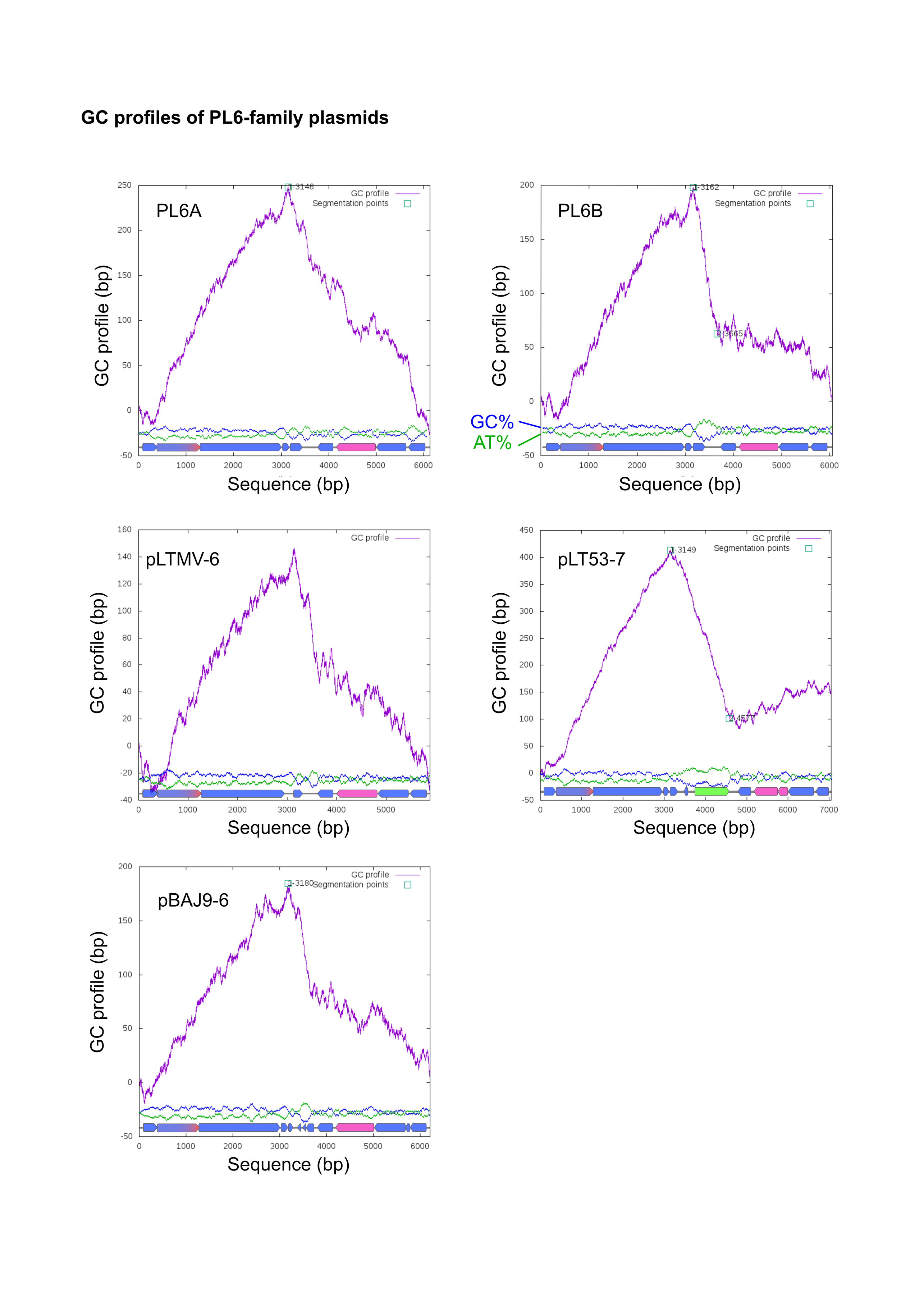


**Legend**: GC profile (Y-axis, purple) versus plasmid length (X-axis, bp) plots were produced using the method of [17] via the webserver at http://tubic.tju.edu.cn/GC-Profile/. Segmentation points are indicated on the plots, except for pLTMV-6 where no segmentation value was returned. Just above the X-axes are gene diagrams of the corresponding plasmid, and immediately above those are plots of GC% (blue) and AT% (green) to indicate how GC content varies along the length of each plasmid.

**Figure S2. Alignments of conserved F proteins of PL6-family plasmids**


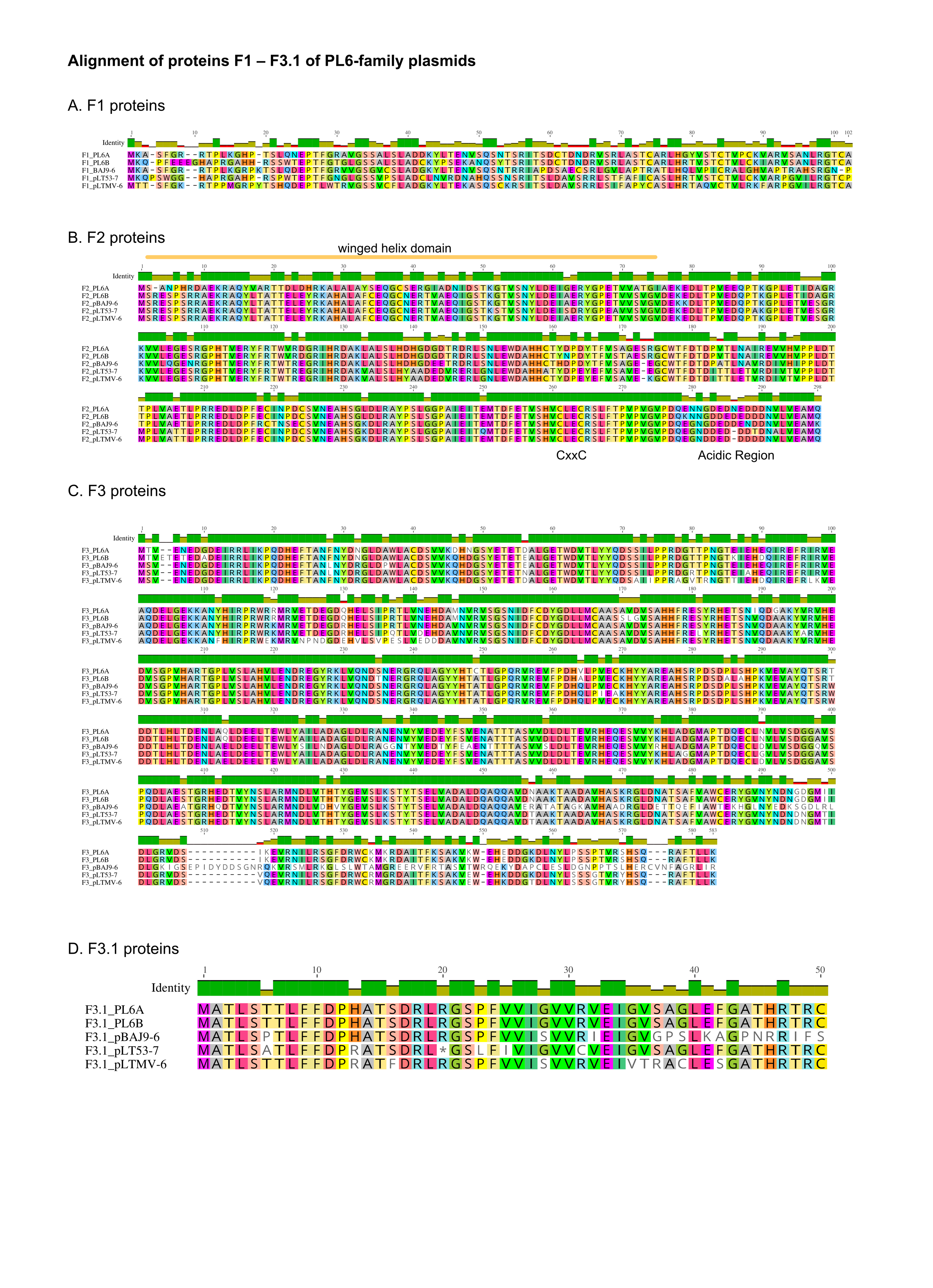


**Legend.** Multiple sequence alignments of predicted F proteins were performed using the Geneious aligner. Topmost level indicates the degree of identity of amino acids at each position (numbers shown above) in the alignment. Amino acids are coloured so as to highlight similarity. Predicted protein domains and features are noted above and below the F2 protein alignment.

**Figure S3. Alignments of conserved R proteins of PL6-family plasmids**


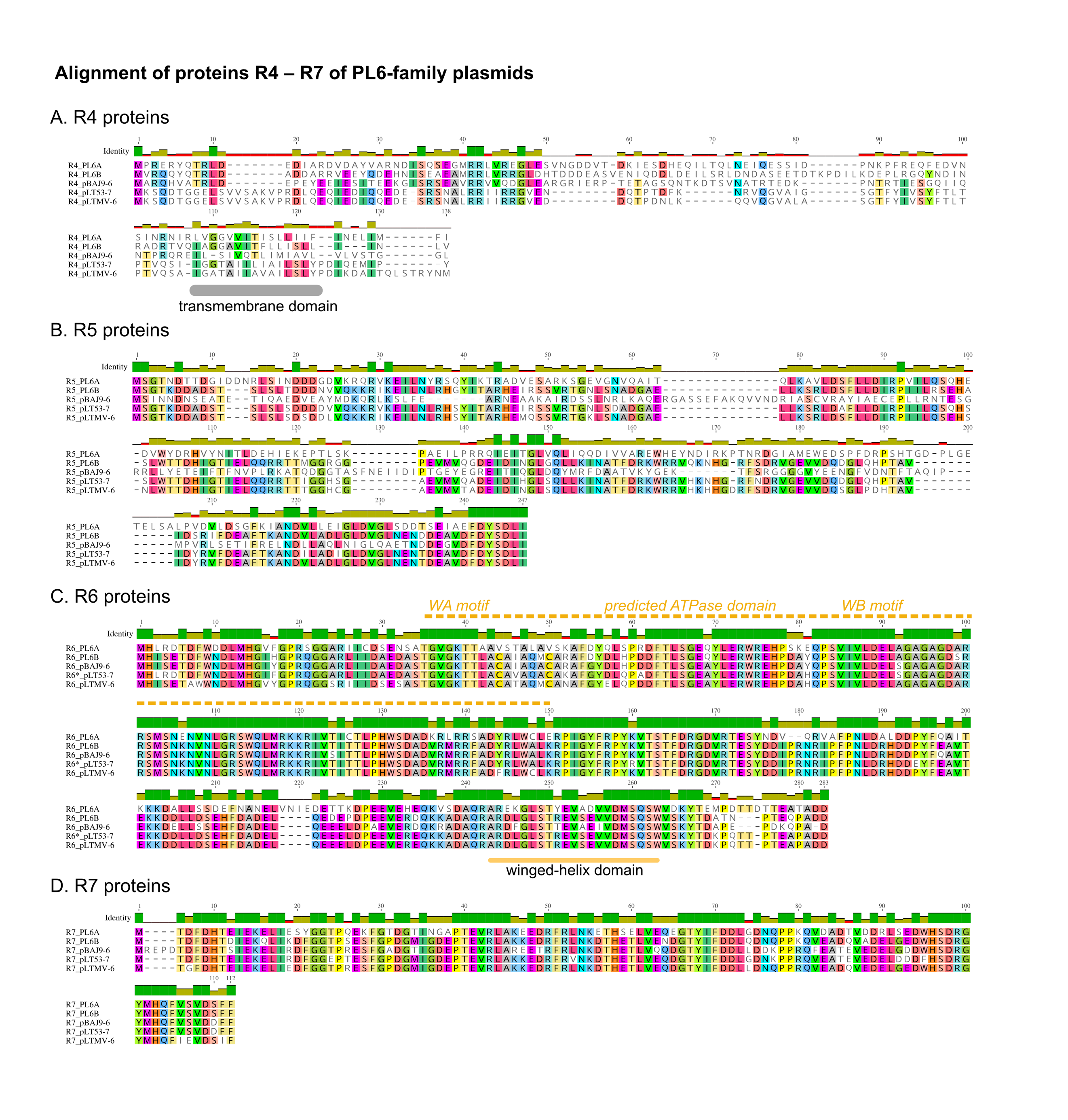


**Legend.** Multiple sequence alignments of predicted R proteins were performed using the Geneious aligner. Topmost level indicates the degree of identity of amino acids at each position (numbers shown above) in the alignment. Amino acids are coloured so as to highlight similarity. Predicted protein domains and features are noted above and below the R4 and R6 protein alignments. The R6 protein sequence of pLT53-7 (labelled as R6*_pLT53-7) was derived by including a -1 frameshift at nt 5889 (within codon 37), which produces an amino acid sequence very similar to the other R6 proteins.

**Figure S4. Phylogenetic tree reconstruction of F3 proteins and relatives**


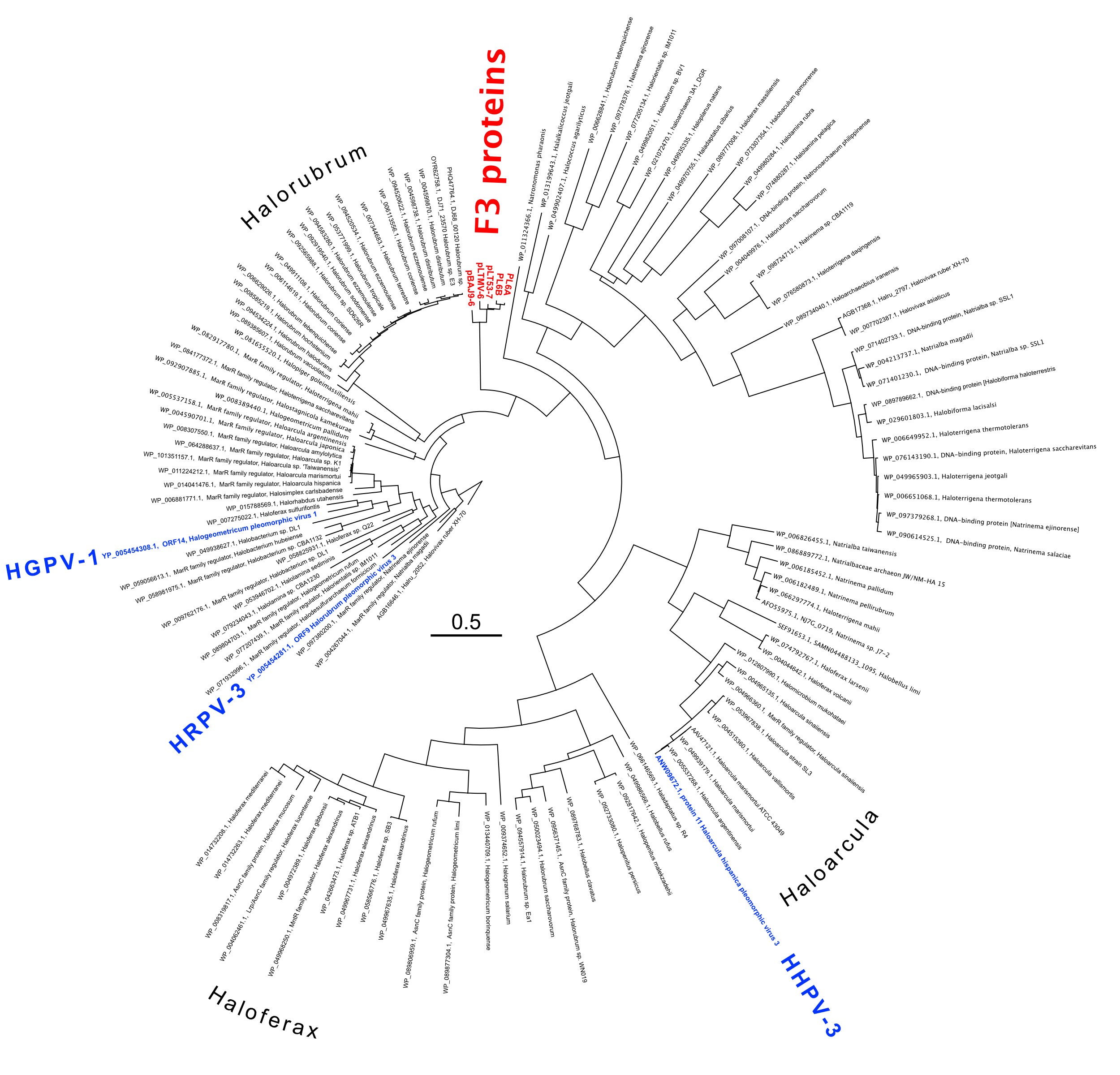


**Legend**. Inferred phylogeny of PL6-family F3 proteins and relatives (RAxML algorithm). Locations of halovirus proteins from HGPV-1, HRPV-3 and HHPV-3 are indicated. Scale bar represents 0.5 expected changes per site.

**Figure S5. Conserved Intergenic sequences (CIS)**


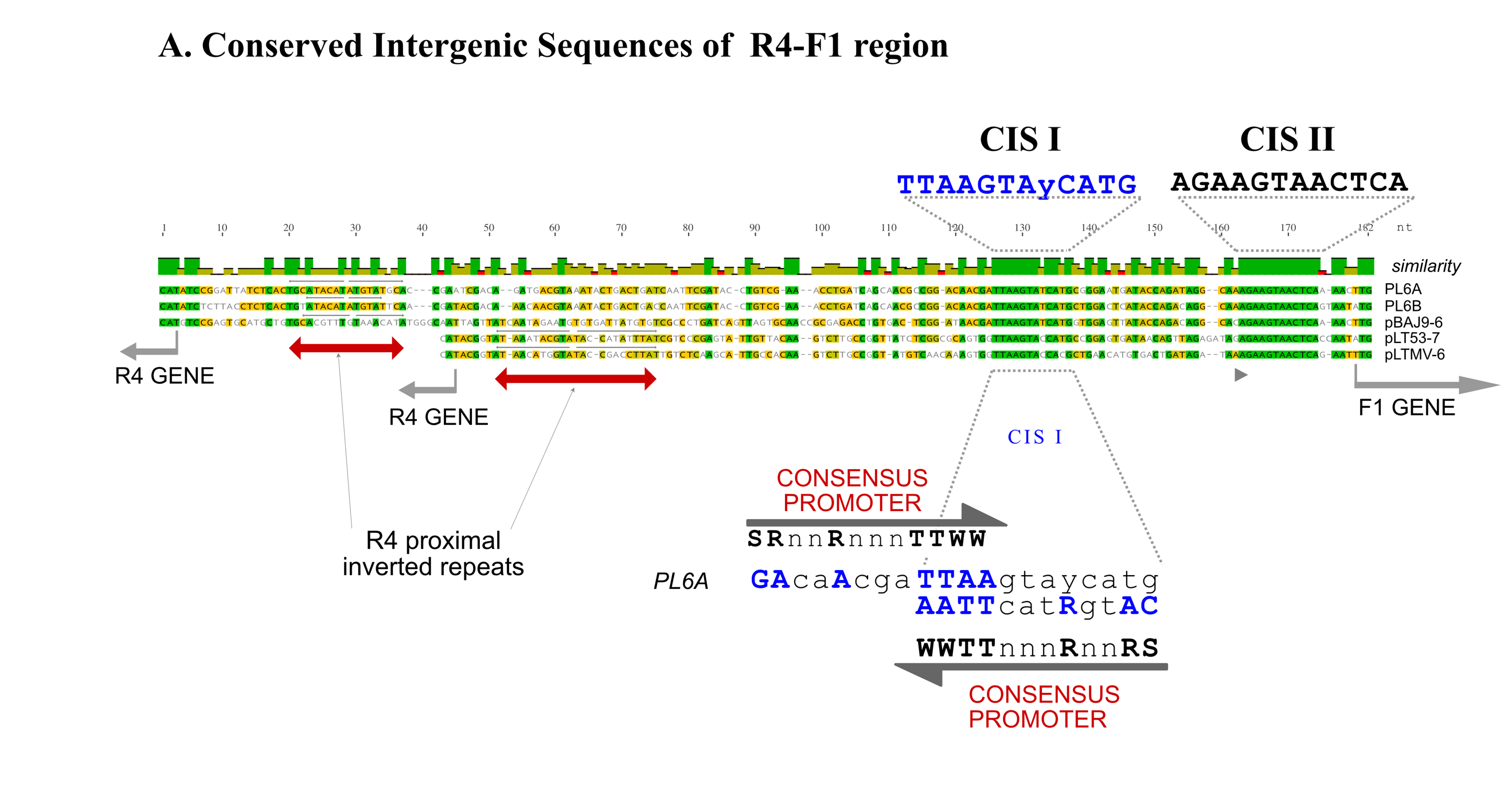


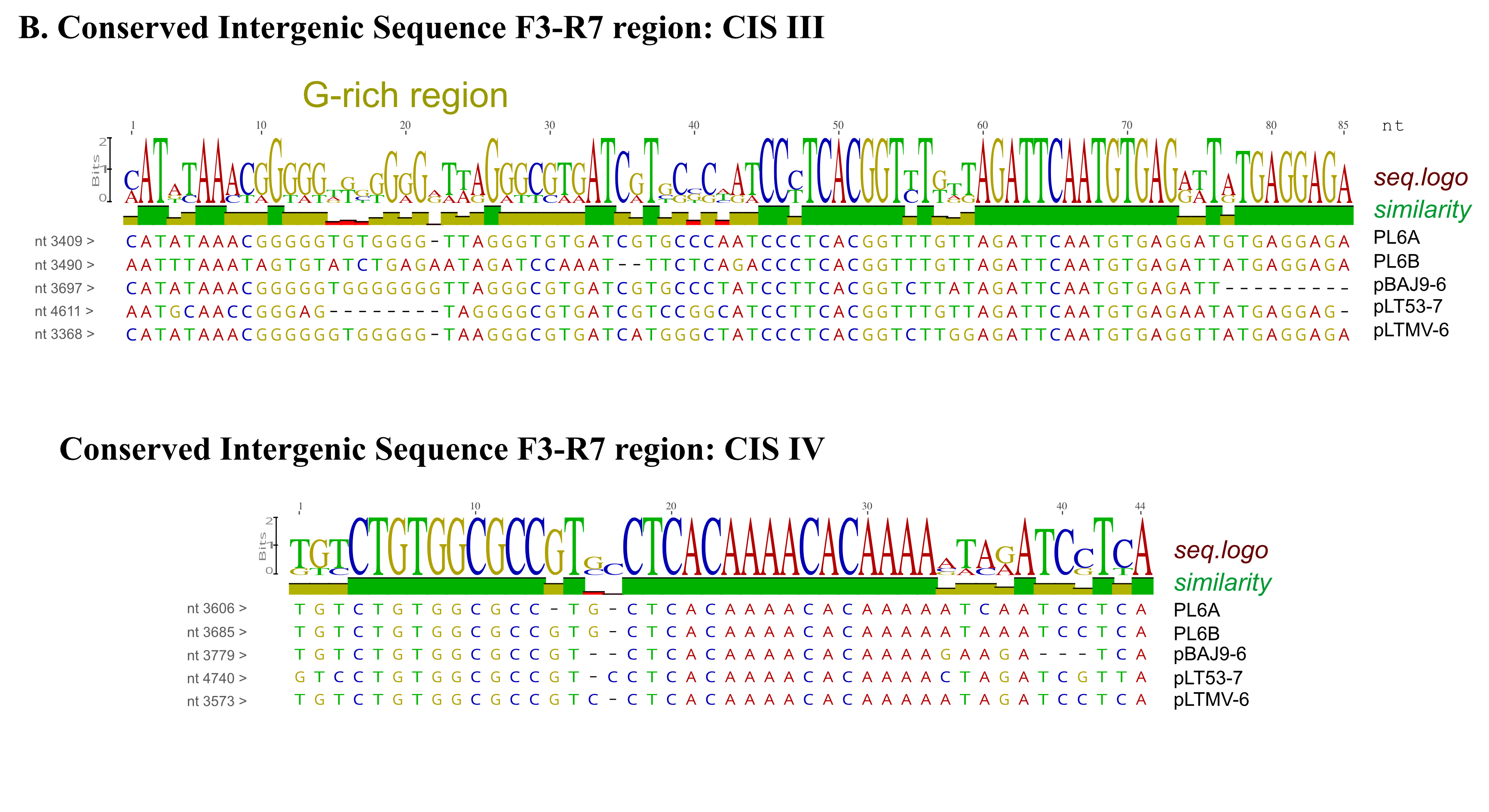


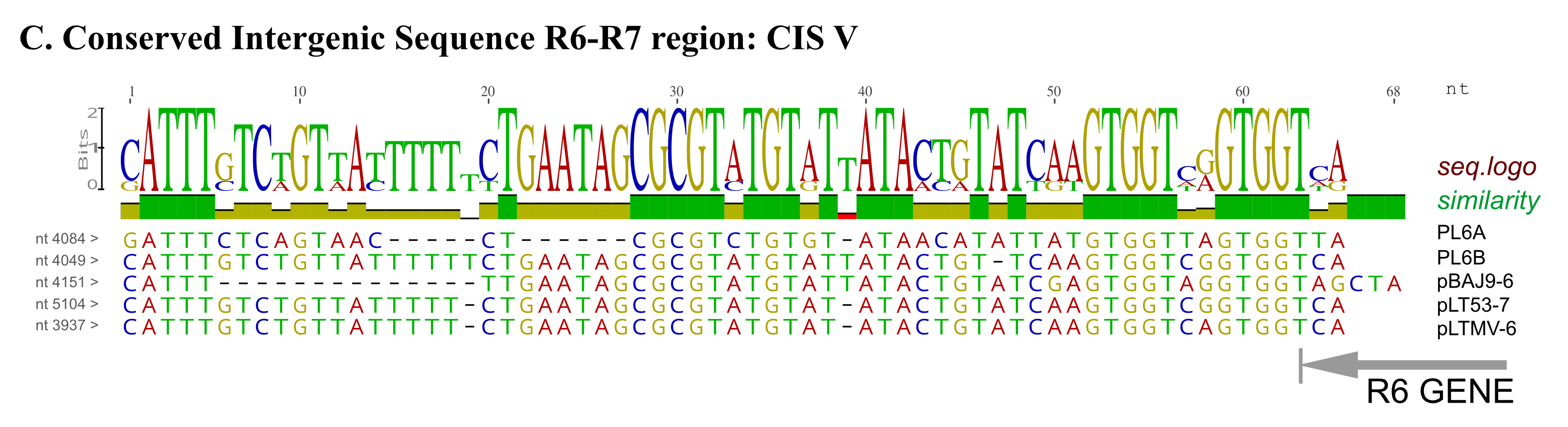


**Legend**: PL6-family plasmid sequences were aligned within the Geneious environment, and strongly conserved intergenic sequences (CIS) were identified and designated CIS 1-V. Plasmid names are shown at the right hand side. Grey arrows show start or end points of annotated genes. Panel A; R4-F1 intergenic region showing CIS I and II, potential promoter motifs and R4 proximal inverted repeats (red arrows). Panels B and C show CIS III, IV and V, identified in the F3-R7 intergenic region (not within F3.1 or F3.2) and in the R6-R7 intergenic region. These also show sequence similarity plots and sequence logos at the top.
